# Enabling Responsible Industry–Academic Collaboration in Mental Health Research and the Involvement of People with Lived Experience

**DOI:** 10.1192/bjp.2026.10817

**Published:** 2026-10-07

**Authors:** Andrew M McIntosh, Sarah Rae, Michael McTernan, Sophie Quick, Lynne Hocking, Ann John, Ed Bullmore

**Affiliations:** 1 Institute of Neuroscience and Cardiovascular Research, University of Edinburgh; 2 UKRI Mental Health Platform PPIE Steering Group, UK; 3 ImmunoMIND Co-production Council; 4 DATAMIND Lived Experience Advisory Group; 5 Swansea University Medical School, Swansea, UK; 6 Institute of Psychiatry, Psychology and Neuroscience, King’s College London

## Introduction

Despite major advances in neuroscience, genetics, and data science, the available psychiatric treatments do not yet provide sufficiently effective or well tolerated options for many people. The global burden of mental illness therefore remains high, underscoring the need for new treatments that better address patient priorities. Meeting this need requires responsible collaboration between academia, healthcare professionals (HCPs), industry, and people with lived experience of mental illness (PWLE). Meaningful and sustainable PWLE involvement is now widely recognised as essential for legitimacy, transparency, ethical sensitivity, building trust, ensuring relevance and acceptability, and helping to align research with the needs and priorities of those it is intended to benefit(1). This perspective is also increasingly being sought by industry and, in the current example, by a large international pharmaceutical company.

In May 2025 we began planning for an in-person scientific meeting between the UK Mental Health Platform, a network of coordinated major investments in severe mental illness research, and an international pharmaceutical company. The aim was exploratory discussion around unmet patient need and future research priorities, involving PWLE, researchers, HCPs, and industry representatives. Despite alignment of purpose and intent, the meeting was arranged and cancelled twice due to uncertainty about regulatory compliance, before finally going ahead as an online meeting in March 2026, hosted by the Mental Health Platform.

This experience provides opportunities to describe some of the challenges that are likely to apply broadly across companies and jurisdictions. We provide several recommendations to those planning future meetings and highlight the importance of proportionate solutions (Box 1).

## Why we need regulations, where they come from, and how they are interpreted

Regulatory frameworks governing interactions between pharmaceutical companies and HCPs were developed in response to past conflicts of interest and have played a critical role in protecting patients, maintaining public trust, and safeguarding scientific integrity. These protections remain essential and were designed to accommodate existing bilateral relationships between industry, academia and HCPs. Critically, current frameworks may not accommodate PWLE involvement in discussions. The challenge is ensuring that regulation continues to serve the public interest while also enabling early-stage, non-promotional collaboration focused on unmet need and responsible innovation. These are conversations that PWLE are well-placed to shape, support and guide.

In the UK, interactions between pharmaceutical companies and HCPs are governed by Association of the British Pharmaceutical Industry (ABPI) guidance. For international meetings, additional national guidance applies, with organisations typically defaulting to the most conservative interpretation where requirements differ. In the case of our proposed meeting, two country frameworks applied. Ambiguities in guidance, combined with differing internal interpretations, led to increased risk perception and delays, despite the absence of promotional intent. The regulatory complexity posed a particular challenge for meaningful involvement of PWLE, especially if they had no organisational affiliation and lacked institutional support to engage with contractual requirements.

In practice, regulatory complexity does not arise solely from differences between national codes. Companies must also interpret how those rules apply to specific situations, and internal compliance teams may reasonably reach different conclusions about the same guidance. This variability can create uncertainty even when all parties are acting in good faith and attempting to remain within established ethical boundaries.

## Problems arising by design and in operationalisation of existing regulations

Specific constraints included limits on meeting size and requirements that company staff be fewer in number than external participants. While defensible from a compliance perspective, these constraints restrict inclusive, multidisciplinary discussion. Such restrictions are particularly challenging in mental health research, where effective dialogue often requires participation from clinicians, PWLE, methodologists and social scientists. Practical requirements may include explicit justification of every participant, clear documentation of the unique contribution each attendee is expected to make, and alignment of those roles with a pre-approved agenda. In some cases, industry representatives cannot outnumber external participants, and any additional company attendees must be formally justified or remain passive observers who are unable to participate in discussion. While these safeguards support transparency, they can make inclusive, multidisciplinary dialogue harder to organise.

Current compliance frameworks appear to be optimised for advisory-board style engagement, where industry seeks defined expert input that is not already available internally. By contrast, early-stage scientific exchange that is characterised by exploratory, “blue-sky” thinking may not fit easily into existing models.

In some circumstances, financial support cannot be provided unless a project has clearly defined deliverables, milestones, and contractual expectations. As a result, exploratory in-person meetings may become difficult to support, even when they are explicitly non-promotional and focused on unmet patient need. When participants include HCPs who prescribe medications, additional safeguards apply to prevent perceived influence on prescribing behaviour. This can narrow discussions to tightly defined, transactional engagements.

Paradoxically, explicit consultancy on named compounds may be permitted, while broader, pre-competitive discussions about unmet need or desired characteristics of future treatments may be discouraged, despite being patient-centred and non-promotional. In our experience the co-presence of PWLE and prescribers was treated as a potential risk, based on concerns about indirect pressure on prescribing. This approach does not fully reflect contemporary mental health research practice, where PWLE involvement is widely regarded as an ethical and scientific necessity rather than a source of undue influence.

Within companies, PWLE and HCP engagements are frequently managed through separate contractual and compliance processes, requiring extensive internal review. This increases administrative burden and slows collaboration. Additional complexity arises because different engagement models (sponsorship of educational events, independent research funding, and formal collaborative agreements) each carry distinct governance requirements and differing levels of permissible industry involvement. This can make it challenging for academic partners to understand which route is most appropriate at early stages of idea development. Relocating meetings to a single country does not necessarily resolve these issues, as similar approval processes and timelines often apply.

These challenges may be particularly significant for PWLE who participate independently rather than through an institution. Unlike academic and clinical participants, PWLE may lack organisational support for contracting, indemnity and payment. Requirements for predefined roles may also sit uneasily with meaningful coproduction. Accessibility needs, meeting logistics and involvement after key decisions have been taken may further limit effective participation.

Online meetings hosted by academic institutions may sometimes represent the only feasible and compliant route for early-stage dialogue, particularly where regulations prevent financial support for exploratory in-person interactions. This approach allows dialogue and scientific exchange to continue but it does not fully substitute for the trust, nuance, and creative collaboration that often emerge during face-to-face meetings and may limit inclusivity. Reliance on these compromises highlights the need for clearer, proportionate frameworks rather than procedural circumvention.

## Towards proportionate frameworks for collaboration

While the underlying regulatory principles remain essential, our experience suggests that existing frameworks may prioritise risk avoidance over facilitation of early-stage, non-promotional collaboration. This may disproportionately affect mental health research, where multidisciplinary exchange and PWLE involvement are central, and may reduce opportunities for companies to address patient need. Importantly, many of these constraints arise from compliance systems designed to prevent inappropriate influence on clinical decision-making. This objective remains essential. The challenge is not the existence of regulation itself but ensuring that frameworks are proportionate to the different types of collaboration now required in modern, multidisciplinary mental health research.

Robust regulation is indispensable to protect patients, maintain public trust, and prevent conflicts of interest. However, regulation must also evolve to reflect contemporary research practice and the urgent need for more effective and better-tolerated mental health treatments.

This is a timely opportunity to develop a flexible framework for industry, people with lived experience, HCPs and academia to work together towards common goals that deliver tangible benefits to people with severe mental illness. Refining existing frameworks to enable responsible, transparent collaboration without compromising public safety or trust will be critical to translating scientific advances into meaningful benefits for people living with mental illness.

Despite these many challenges, the meeting was finally able to proceed with all parties represented. The event was valuable both to the company and to the participating PWLE, who have subsequently engaged in follow-up meetings. The company’s interest in further meetings focused specifically on lived experience illustrates the value that can be placed on PWLE perspectives in the development of future therapies. PWLE representatives within the Mental Health Platform are keen to work with industry in pursuit of more effective and better-tolerated treatments. We should therefore seek to enable the ethical involvement of PWLE in industry collaborations and develop proportionate approaches that are fit for purpose.

## Box 1 Recommendations for responsible collaboration

### For those planning future meetings

- **Support multidisciplinary inclusion**
  Ensure meeting size and structure rules allow meaningful participation from relevant disciplines, prioritising need for representation and involvement over arbitrary thresholds.
- **Maintain transparency and documentation**
  Clear agendas, declarations, and governance should remain central to all engagements.
- **Enable pre-competitive exploratory conversation**
  Clarify pre-competitive engagement providing clear pathways for early, non-promotional scientific discussion before formal project milestones are defined, supported by transparent governance.
- **Provide practical support for PWLE**
  Provide contracting and payment support for PWLE and particularly those with no institutional affiliation.

### For policymakers (companies, government and regulatory authorities)

- **Recognise PWLE involvement as a public-interest safeguard**
  Treat meaningful PWLE involvement as integral to ethical and relevant research, rather than as a compliance risk.
- **Align guidance across organisations and jurisdictions**
  Greater national and international consistency would reduce uncertainty and delay while maintaining high standards.
- **Reduce variability in regulatory interpretation**
  Develop clearer operational guidance to minimise divergence in interpretation across organisations and countries.
